# Medico-Chirurgical Transactions. Vol. XXV

**Published:** 1843-01-01

**Authors:** 


### Medico-Chirurgical Review,

### No. LXXV.

**[No. 35 of a Decennial Series.]**

OCTOBER 1, 1842, TO JANUARY 1, 1843.

MEDICO-CHIRURGICAL TRANSACTIONS, Published by the Royal Medical and Chirurgical Society of London. Volume XXV. London: Longman and Co. 1842.

THE present volume of the Society's Transactions is not distinguished by any elaborate contribution, but contains a large number of valuable cases, and many interesting observations. The Society, indeed, is daily growing in usefulness and reputation. Like Aaron's wand, it has swallowed up its rivals, and the other medical societies of London are fast disappearing from its side. Admitting discussions as well as publishing papers, comprising within its ranks all that is distinguished and honourable in the Profession, it has attracted to itself talent as well as numbers, and is now sailing on a full tide of popularity and success. Long may it do so.

The Contents of the volume before us are as follows :—

1. Case of cyanosis, depending upon transposition of the aorta and pulmonary artery ; by W. H. Walshe, M.D. Communicated by John Forbes, M.D.—2. Case of aneurism of the ascending aorta, bursting into the right ventricle ; with a communication between the two ventricles ; by Thomas S. Beck. Communicated by Robert Lee, M.D.—3. On the structure and functions of the human placenta ; by John Dalrymple, Esq.—4. On the relation between the symmetry and the diseases of the body ; by James Paget, Esq.—5. An account of a case of extensive disease of the pancreas ; by J. A. Wilson, M.D.—6. Remarks on typhus fever ; by John Bostock, M.D.—7. Cases of laryngitis, relieved by operation ; by John Wilson, M.D.—8. Peculiar symptoms affecting an entire family, and terminating in death ; by the same.—9. A case of congenital cataract, where sight was acquired by operation, at the age of twenty-three years ; by R. A. Stafford Esq.—10. On diseases which affect corresponding parts of the body in a symmetrical manner ; by William Budd, M.D.—11. Notice of cases of plague contracted in the Lazzeretto of Constantinople, in a letter addressed to Dr. Davy ; by M. Antoine Pezzoni. With Remarks by Dr. Davy. Communicated by Thomas Hodgkin, M.D.—12. Observations on tubercle of the brain in children, with a tabular view of thirty cases of the affection ; by P. Hennis Green, M.D. Communicated by Dr. Burgess. —13. Case of local tubercular deposit upon the surface of the brain ; by

Robert Dunn, Esq.—14. A case of stricture of the trachea; by W. C. Worthington, Esq. Communicated by James Copland, M.D.—15. History of a remarkable case of tumors, developed on the head and face; accompanied with a similar disease in the abdomen; by Henry Ancell.—16. Account of a case of irregular formation of the heart, accompanied with a supernumerary valve in the pulmonary artery; by Theophilus Thompson, M.D.—17. Notes of a case of petechial cow-pox, with observations on the development of the hæmorrhagic diathesis; by George Gregory, M.D.—18. On acute ulceration of the duodenum, in cases of burn; by T. B. Curling, Esq.—19. Cases of malformation of the heart; by T. B. E. Fletcher, M.D. Communicated by Richard Partridge, Esq.—20. Observations on a particular form of encysted tumor, which occurs in the neck, but is not necessarily connected with the thyroid body; by Benjamin Phillips, F.R.S.

We shall take these Papers in succession.

### I. CASE OF CYANOSIS, DEPENDING UPON TRANSPOSITION OF THE AORTA AND PULMONARY ARTERY. By *W. H. Walshe,* M.D.

Dr. Walshe was prevented from learning much of the state of the child during life. The following are the particulars of the case.

*Case.*—Dr. W. saw the child, a male, aged ten months, decrepit, in a paroxysm of frequent but not periodical occurrence. In such a fit, the child died two days afterwards, death being hastened by slight diarrhœa and pulmonary catarrh.

"The skin is of deeply leaden colour, particularly at the toes and extremities of the fingers: the surface feels cool, and the infant appears to suffer from chilliness. The leaden discoloration of the face is general, but particularly deep at the upper lip and both internal canthi: no particular pulsation in these situations. The infant throws about its arms constantly, and is much agitated; the eyes appear prominent and their expression staring; respiration very frequent and somewhat gasping; pulse between 120 and 130; no abnormal murmur in the cardiac region, or in the course of the great vessels; heart's action tumultuous, impulse strong and widely diffused." 2.

*Examination after Death.*—About half an ounce of pale serosity in pericardium. *Heart.* "Position in the chest natural; surface blueish, from distension of the large and small veins with blood; apex slightly twisted to the left, from the accumulation of coagula in the venæ cavæ and right auricle, is formed by the extremity of the right instead of the left ventricle. The right half of the organ lies anterior to the left. The aorta rises from the right ventricle, and at its origin slightly overlaps the pulmonary artery, which springs from the left ventricle: no communication (except by the ductus arteriosus) exists between these vessels. The aorta contains a good deal of black grumous blood, intermixed with a few fibrinous granules; its lining membrane unstained and healthy. From the upper border of the arch rise two subclavian and two carotid arteries; the two coronary arteries are given off in the usual way immediately above the sigmoid valves; the latter, as well as those of the pulmonary artery, are of the usual number, smooth, transparent and healthy. The ductus arteriosus, pervious and wide enough to admit with ease a good-sized probe, rises from the posterior border of the pulmonary artery about an inch and a half above the origin of that vessel. The opening into the aorta is of oval shape and exactly opposite the origin of the left subclavian; an elevation of the lining membrane of the vessel is manifest along its lower border. The walls of the ductus arteriosus are slightly thickened, tough and indurated (commencing conversion into ligamentous tissue). The right auricle, containing an enormous quantity of black grumous blood, which pours from both venæ cavæ when divided, receives those vessels in the usual manner at its posterior surface. Close to the appendix is a small coagulum of shredy fibrine, firmly adherent by prolongations between the interstices of the musculi pectinati; the adhesion in one place so intimate, that the coagulum will not separate without being torn. Left auricle: Walls almost membranous, scarcely any muscular fibres to be seen in them; from general appearance its size may be estimated at about one third of that of the right; it receives the four pulmonary veins in the usual way; these vessels contain blood of the kind already described. Auricular septum: Foramen ovale perfectly open, of oval form, presents its greatest diameter from above downwards: measures about three eighths by two eighths of an inch (Engl.),—when stretched slightly, four by three eighths. Its anterior border is valvular, semilunar, thick, firm, and has an opaque whitish appearance under the endocardium; the posterior is thinner and sharper. There is another, but minute, opening in the septum, capable of admitting a small probe. Right ventricle furnished with columnæ carneæ of large dimensions, especially with one of unusual size; endocardium healthy, auriculo-ventricular orifice free, provided with a mitral valve, which is smooth, transparent and thin. Left ventricle: Scarcely any columnæ on its surface, all of them very ill developed; endocardium healthy: auriculo-ventricular orifice free, and provided with a healthy tricuspid valve, one division of which, however, is extremely small. Ventricular septum is not perforated."

*Lungs.*—Venous engorgement where pulmonary veins issue from them—several emphysematous vesicles here and there.

*Liver* congested with fluid blood. Umbilical vein impervious—ductus venosus presenting a speck of blood in centre.

*Spleen* gorged with blood. *Kidneys* congested also.

*Vena Cava Inferior* enormously distended, especially close to the liver, from accumulation of grumous, semi-coagulated blood.

*Cranium* not opened.

Dr. Walshe deduces from the preceding case, and a comparison of it with others that have been recorded, the following series of inferences:—

1. "The transposition of the primary arterial trunks was attended with similar transposition of the ventricles. The right ventricle presented the muscularity characteristic of the left; the left the thinness of wall natural to the right. That this was not a mere result of the comparative functional activity would appear from the interchange of valves,—the right having the mitral of the left, and the left the tricuspid of the right side. The position even of the ventricles, both as regards their relation to each other and to the chest generally, was changed; the plane of the right being anterior to that of the left, and its point forming the rpex of the heart. The aorta, too, slightly overlapped the pulmonary artery at its origin.

2. "The aortic system constantly circulated black blood, with the exception of the extremely small quantity of red carried from the pulmonary artery by the ductus arteriosus.

3. "The pulmonary artery constantly circulated florid blood, with the exception of the small quantity of black which may have found its way through the foramen ovale from the right into the left auricle.

4. "The heart was hypertrophous.

5. "The viscera generally, so far from being smaller, were actually rather larger than in naturally conformed individuals of the same age, though more flaccid (with the exception of the liver) than usual; the muscular and adipose systems were defectively nourished, but not in any very extraordinary degree.

6. "Hence it follows, that the muscular system and viscera may be nourished, without important derivation from the normal state, by blood of which a minute part only is oxygenised. In other words, an individual may continue to live and be nourished in a state of partial asphyxia, when this has existed from birth.

7. "But as the blood in the portion of the aorta lying between the opening of the ductus arteriosus, and the heart, was wholly venous, and as the coronary arteries rose from the aorta in the usual position close to the sigmoid valves, it follows that the nutrition of the heart must have been wholly effected by non-oxygenated blood. Tiedemann has suggested that in the case observed by him the bronchial arteries might have been the instruments of partial arterialization. They were in that instance 'unusually large,' and he supposes that the corresponding veins brought back their contents oxygenised to the vena cava and right side of the heart, whence those contents passed by the aorta and coronary arteries to its substance. Admitting this to be correct, (though I am not satisfied that the notion of arterialization occurring in the bronchial arteries is easily admissible,) the quantity of red blood finding its way to the tissues of the heart must have been so small, that the admission scarcely alters the state of the case.

8. "In the present case, and in the only three others in the descriptions of which distinct reference is made to the point, the pulmonary and cavæ veins opened respectively into the left and right auricles, as in the natural state: it may be inferred from the existence of cyanosis that they did so in other recorded cases also.

9. "This malformation has a tendency to associate itself with others;—patency of the foramen ovale, and a pervious state of the ductus arteriosus. The mode of origin of the brachio-cephalic trunks from the arch of the aorta in two of the four cases where the point is mentioned, was also irregular: there were two only in Baillie's case, four in that now described.

10. "This pervious state of the ductus arteriosus, which, under other circumstances, would have caused admixture of black blood with the general red current, has, when the present malformation exists, precisely the contrary effect: upon it the whole system depends for its supply of arterialized blood.

11. "The difference existing in this case in the width of the two auriculo-ventricular orifices was of the same kind as in the natural condition; that is, the width of the right orifice exceeded that of the left: and the width of the pulmonary artery at its origin exceeded that of the aorta, as in naturally-formed hearts. Hence, in respect of relative width, the auriculo-ventricular orifices had undergone no transposition, whereas the contrary was the case with the arterial orifices: in other words, while the two orifices of smaller and larger size are respectively, in the natural state, on the same side of the heart, a small and a large one existed on each side in the case described.

12. "The effect of this on the circulation must have been that a less quantity of black blood was sent into the aorta at each systole than that proportional, in the ordinary state, to the quantity received into the right ventricle: and a greater quantity of arterialized blood was driven at each systole into the pulmonary artery than that proportional, in the natural state of things, to the quantity received into its corresponding ventricle.

13. "The pulmonary lesions, vesicular and interlobular emphysema, must have materially affected the suffocative paroxysms; and these were, in turn, most probably the original cause of the emphysema, more especially of the interlobular variety.

14. "In addition to the usual attendants upon cyanosis, deficient calorification, dyspnœa and suffocative paroxysms, the three infants who lived for any time (those observed by Langstaff, Farre, and myself,) suffered under diarrhœa more or less habitual.

15. "In seven cases in which a fœtus thus conformed was born alive, the duration of life was as follows:—

"About two months. (Baillie.)
"Ten weeks. (Langstaff.)
"Five months; the infant died of variola, caught from a sister. (Farre.)
"Twelve days. (Tiedamann.)
"Four or five days. (Dugès.)
"Ten weeks. (Martin.)
"Ten months. (Present case.)

16. "The liver was very considerably enlarged: may it, from its augmented size, have been enabled to effect a greater than the physiological amount of decarbonization of the venous blood, and thus have contributed in some measure to diminish the necessity for arterialization in the lungs?

17. "The decrease in the intensity of the blue discoloration of certain parts after death, shows of itself that this discoloration depended upon the circulation of black blood in the arteries.

"I may observe, too, that the aorta contained black semi-coagulated blood,—that the examination of the body did not take place until thirty-five hours after death,—and the weather at the time was warm and damp: yet there was no discoloration of the lining membrane of this vessel. The case consequently supports the doctrine established by M. Louis in respect of aortic staining,—namely, that this anatomical state requires for its production some as yet unascertained change of the arterial tissues or blood, in addition to the physical conditions associated in the present instance." 14.

The preceding inferences are interesting and just. They admit of no abbreviation, and it were a loss to our readers to omit them.

### II. Case of Aneurysm of the Ascending Aorta, bursting into the Right Ventricle: with a Communication between the Two Ventricles. By *Thomas S. Beck,* Lecturer on Surgery at Sydenham College.

The patient, a surgeon, aged 31, had the usual symptoms of aggravated cardiac disease, conjoined with the following auscultic signs:—"Dulness on percussion over the whole of the chest. Vesicular murmur distinct, yet diminished in intensity and mixed with bronchial *râles.* The sound of the heart's action heard over the whole anterior and posterior parietes. The impulse increased. Both sounds of the heart were distinctly audible, and following the second sound, a continuous, very superficial, sawing sound with tremor, most distinct at the base of the heart, near to the sternum. This sound was very disagreeable to the ear, being so distinct and apparently close to it. It was continuous throughout the whole of the action of the heart, but loudest directly after the second sound."

Anasarca, ascites, and hydrothorax, closed the scene.

*Examination.*—Passing over the condition of the lungs, of which it is merely necessary to say, that there were congestive and pleural effusion, we arrive at the *Pericardium,* in which were three or four ounces of red-coloured serum.

"The *Heart* was much enlarged: taking the fist of the subject as an approximate measure, it was more than 2½ times that size. After removing it, laying open the right ventricle and part of the ascending aorta, and washing with a stream of water, the lining membrane presented a thickened pearly white appearance, particularly marked near the orifice of the aorta, the right and left semi-lunar valves were thickened, whilst the posterior had undergone little change. On the valves, and at the commencement of the aorta, were several opaque spots. In the centre of the right valve existed an osseous deposit, beginning at the corpus aurantii, and extending to the walls of the ventricle, so as to keep it constantly distended, and although the action of the other valves was impaired, yet they were sufficiently free as to prevent regurgitation, except to a slight extent. The right sinus of Valsalva was enlarged, and presented a round open communication between the aorta and right ventricle, sufficiently large to introduce the end of the little finger. On laying open the right ventricle, the lining membrane presented the same white appearance, though in a less marked degree, the valves were little changed from their natural state, whilst immediately beneath them lay the collapsed sac of an aneurysm, resembling the end of a finger of a glove, about three-quarters of an inch in length, and bursting in the extremity in a large ragged opening with two small openings at the side, the edges of all of which were worn and rounded as if the blood had passed for some time by these openings. The mitral valves were healthy, excepting a white tinge in their colour. No coagulum was found in the sac. The right side of the heart was filled, but not distended, with coagulated blood. Immediately at the base of the sac there existed a communication, the size of a goose-quill, between the two ventricles. There was no communication between the auricles." 20.

Mr. Beck thinks there can be little doubt that the opening between the aorta and right ventricle was the remains of an aneurysmal sac; and this certainly does seem to be most consistent both the morbid appearances and history.

### III. On the Structure and Functions of the Human Placenta. By *John Dalrymple,* Esq.

Mr. Dalrymple has found, since the prosecution of his researches on this subject, that his conclusions agree remarkably with those of Weber, drawings to illustrate which are given in Wagner's Physiology.

Mr. Dalrymple sets out with asserting, that any direct communication between the uterine vessels, and those of the placenta is impossible. He proceeds :—

1. "The umbilical arteries, after dividing and passing in a convoluted and serpentine form over the fœtal surface of the placenta, dip at various intervals into its substance, there dividing and sub-dividing infinitely. The trunks are covered on the surface of the organ by the fœtal membranes, and each branch, as it dips into the thickness of the tissue, carries before it a fold of the chorion.

2. "The whole mass of the placenta is made up of the innumerable ramifications of the arteries, terminating in beautiful coiled and convoluted capillaries, which form tufts or bouquets at various intervals; these finally become continuous with the minute origins of the umbilical vein, which returns to the fœtus in the same direction that the arteries left it, viz., coiled and twisted in the umbilical cord.

3. "The vein and its branches are greatly larger than the arteries and their subdivisions, but less numerous.

4. "The direction of the branches of the umbilical arteries is from the fœtal to the uterine surface of the placenta, passing obliquely from the centre to the circumference and edges. The veins return in the reverse direction. All the vessels, besides their own proper coats, are enclosed in a fold of chorion.

5. "As the minute branches of the arteries terminate in serpentine and very intricately coiled capillaries, so are these latter subdivided into masses, or tufted and bouquet-like processes, clothed by prolongations of the before-mentioned membrane.

6. "This membrane (chorion) constitutes (by division into processes) true villi, and each villus contains a tortuous capillary, which entering from the arterial side leaves it by the venous: as the vessel leaves the villus there is a slight but manifest increase of size.

7. "A single tuft or collection of villi, well injected, and laid flat under an inch, or half inch object-glass, appears at first sight an inextricable confusion of curiously-contorted capillary vessels; but separated by needles, and a single villus detached, or expanded beneath a higher magnifying power, this seeming confusion is reduced to order, and the true anatomy of these vessels explained.

8. "The membrane enclosing the vessels and capillaries is studded on the exterior by nucleated cells, resembling an irregular epithelium.

9. "The enclosed tufts, or capillaries, nowhere anastomose with other than fœtal or umbilical vessels.

10. "The arteries and veins, though covered by a common membrane, are nowhere so closely bound together, as to constitute one undivided though really double vessel, as described by Dr. Reid, and the 'blunt extremities,' adverted to by that gentleman, appear to me to be the villi of the placenta.

11. "The villi are not connected together by cellular tissue, but the mass of the placenta is made up by the vascular divisions and subdivisions, and by the tufts or bouquets of capillaries; the interstices are everywhere free, and communicate with each other.

12. "There are no distinct or defined cells constituting a maternal portion of the placenta.

13. "The uterine surface of the organ is covered by the decidua, which does not appear to enter further than between the lobules, and the depth to which it thus penetrates varies with the extent of the fissures.

14. "Stretching from the fœtal to the uterine surface of the placenta are irregular semi-fibrous bands, more firm towards the fœtal surface, and nearly disappearing towards the decidual: these appear to give firmness to the spongy mass, and to a certain extent support to the tufts of villi.

15. "The bouquets of capillaries are found in all parts of the placenta, but are more numerous at the uterine surface, where they will be found close beneath the decidua.

16. "Upon the decidual surface may be observed, thinly scattered, certain papillæ, somewhat obtuse and blunted, about a line and a half in length, which seem to be constituted by innumerable coiled and minute capillaries. Are these the analogues of the fœtal cotyledons?" 25.

Mr. Dalrymple makes some observations explanatory of the differences, unfortunately too numerous and wide between microscopical observers. Perhaps they rather account for those differences than are likely to end them.

He explains too the placental circulation thus :—The maternal blood *may* be extravasated by "the curling arteries" into the spongy mass of the placenta—then the fœtal tufts become, in function, absorbent villi, taking up the nutrient part of the fluid, which is carried to the fœtus by the umbilical vein. This blood again is in part returned to the placenta by the umbilical arteries : part however is retained, and appropriated to the nourishment and growth of the embryo.

"In the placenta must go on a double action, or interchange of fluids; for the blood returned to this organ by the arteries is unfitted for a second circulation through the embryo : at least this is true in part if not entirely. Hence, while the blood, or nutrient material of the blood, brought by the uterine arteries, and previously aërated by the mother, enters by endosmose the absorbent capillaries of the fœtal villi, that portion of the fœtal blood that requires the action of oxygen escapes by exosmose, and returns by the uterine sinuses and veins to the maternal heart. Thus the lungs of the mother are in fact the lungs of the the fœtus, and hence the comparatively simple system of the vessels of the cord." 27.

Mr. Dalrymple's industry and accuracy are well known. We trust that the researches of others will confirm his.

### IV. ON THE RELATION BETWEEN THE SYMMETRY AND THE DISEASES OF THE BODY. By *James Paget*, M.R.C.S.

Mr. Paget thinks it highly probable that it is a law of the animal economy, that, when uninfluenced by disturbing causes, all general or constitutional diseases affect equally and similarly the corresponding parts of the two sides of the body.

Thus he found in the body of a woman exactly the same kind of disease in each elbow-joint. In another woman, aged 70, he found exactly similar changes in each knee-joint. In another person both hip-joints were so affected. The same thing occurred in another instance. He mentions two more knee-cases, and one humeral one. Then he has got a pair of hind legs of a dog, and two ovaries of a woman to exhibit.

Mr. Paget quotes, to support him, the researches of M. Bizot. "He found that in 2,171 cases of yellow spots in the arteries, a symmetry of the morbid changes was wanting only 62 times; that, in 659 cases of lesions consecutive to such spots, it was wanting only 51 times ; and that many of even these few exceptions were connected with an absence of symmetry in the affected arteries, or some similar modifying circumstance."

And after some ingenious, though perhaps not very satisfactory reasoning, Mr. Paget concludes that there may be at least three different conditions in which diseased changes are symmetrical.

"In a first class of cases, they are the result of the gradual degeneration of the tissues in the course of time, or after their functions have ceased, or when, through some general disorder in the economy, the whole body fails of being duly nourished. Such are emaciation, the changes of old age, &c.

"In a second class, the symmetrical changes are the result of a morbid condition of the blood, in which some new material bears a peculiar chemical or organic relation to the whole or a part of some symmetrically-arranged tissue or organ, so that when they come in contact, the mode of nutrition in the tissue is altered, or the new material is deposited in it. These changes are symmetrical, because the same morbid material acts similarly with all similar substances. They are symmetrical and general, when the whole of the seemingly similar tissue has really the very same structure and other properties. But, more commonly, they are symmetrical and local, because the corresponding parts on the opposite sides of the body are the only parts in which the symmetry is, in respect of every property of the tissue, perfect. To this class belong the rheumatic, gouty, scrofulous, tuberculous, cancerous, medullary, and some other symmetrical diseases.

"In a third class the symmetrical changes are the consequences of diseases passing by metastasis from one part to the exactly corresponding part on the opposite side. In some of these a morbid condition of the blood exists, in others it probably does not. In all, I believe that the influence which determines the situation occupied by the diseased process after metastasis is one conveyed from the part first affected through its nerves (which are in a state of morbid organic excitement) to the nervous centres, and thence reflected and conveyed through *its* nerves to the part secondarily diseased. To this class must be referred the metastatic affections of the eyes, tonsils, testes, and probably some cases of rheumatism and gout." 41.

We agree with Mr. Paget, that if symmetry in disease *be* a law, there is probably none in science, to which the exceptions are so numerous. In fact the exceptions may be fairly styled the law, and the law the exception.

### V. An Account of a Case of extensive Disease of the Pancreas, By *James Arthur Wilson,* M.D. Physician to St. George's Hospital.

Dr. Wilson has contributed a very interesting case. It is that of a gentleman's servant, aged 41, a hard liver, admitted into St. George's, Oct. 21, 1835. He looked unhealthy and distressed. "He had long suffered from a constant pain in the epigastrium, (described as a 'drawing and pulling together of the pit of the stomach,') which was occasionally aggravated by paroxysms into severe agony. It was felt most in the recumbent posture, after taking food, and when the bowels were constipated. When most severe, it was accompanied by headache, giddiness and sickness. He had vomited blood sixteen months before his admission, and frequently during his illness. The bowels were obstinately costive, the urine was free. He occasionally complained of a 'fluttering' in the region of the heart, but there was no irregularity in the pulse, which was low and slow, averaging about sixty-five beats in the minute. He was much distressed by 'cold feet,' and complained greatly of want of sleep." About a month after admission, the pain suddenly returned in the epigastrium with augmented intensity. It was ushered in with shivering and violent headache and sickness. Maniacal delirium followed, coma succeeded and in that the patient died. During his illness, no relief was obtained from any but active aperient medicines.

*Examination of the body.*—There were universal adhesions of the pericardium, but no disease of the heart. The brain was softer than usual, with considerable vascularity of its medullary structure; some clear serous fluid was observed on the outer surface of the arachnoid membrane, but very little had been effused into the ventricles. The liver was pale, soft, and friable. The spleen was very pulpy.

"The pancreas was of unusually hard texture, and much contracted in its general dimensions. Its ducts were universally filled with a compact, white, earthy deposit, which, on analysis, was found to consist of nearly pure carbonate of lime, with a fibrinous nucleus of animal matter." 45.

### VI. CASES OF LARYNGITIS RELIEVED BY OPERATION. By *John Wilson*, M.D. Physician to the Middlesex Hospital.

Dr. Wilson makes some very just reflections, which are not, perhaps, so widely acted on as they might be.

"Generally," he observes, "as the difficulty of respiration increases in laryngitis, so does the danger. The primary obstruction arises from the contraction of the rima glottidis, the secondary from a less quantity of air being thus permitted to pass into the chest. The lungs become only partly expanded, on inspiration, and this deficiency is sought to be supplied by the number of respirations being increased. Thus, as the circulation of air becomes embarrassed, the circulation of fluids becomes so likewise. Hence blood or serum infiltrates into the tissue of the lungs where the respiration is most defective; as this infiltration increases, the lungs become less permeable to air, while the other parts, yet enabled to maintain their integrity, having now to discharge the whole respiratory functions of the lungs, become over-exerted, and ultimately portions of these, most commonly the margins, become distended with air, and unable to contract. Thus do the emphysematous, like the œdematous portions, become deprived of their contractile power; the permeable parts becoming further diminished, and the lungs less able to perform their office: while at the same time the bronchial irritation is increasing the danger.

"Lastly, the blood, from its imperfect circulation through the lungs, becomes vitiated by defective oxygenation; then its action on the brain produces coma, while its dark veinous colour becomes apparent in the livid countenance.

"These are the immediate precursors of death. Yet this is the stage which the patient is not unfrequently allowed to reach, before it is judged adviseable to make an artificial opening for a freer passage of air; and when then made, death soon follows the operation, and sooner still when blood from the wound descends by the trachea into the lungs.

"Yet in cases where the breathing becomes equally alarming, but where the permeable portions of the lungs may be still sufficient for sustaining life, till time be given (by an operation) for the reparation of the lesions of the other portions, and likewise of the larynx, which then acquires a comparative state of repose,—under such circumstances an operation may never be too late, and recovery may take place, even though respiration may have ceased, if life be not extinct.

"Likewise, although the lungs may have sustained lesions, which must ultimately become fatal, life may yet be prolonged though not saved by an operation. Therefore, in cases where such severe lesions may be anticipated, the proper time for operating should be before they become irremediable.

"It should always be borne in mind, that as the time is prolonged for the effects of other remedial agents, the chance of success for this one is proportionably diminished.

Such also were the reasons that first induced me, in the early stage of acute pleuritic effusion, to have a trochar passed into the affected side of the chest, and to draw off at once nine pints of clear fluid;—thus averting threatened death, and at the same time allowing the lung to expand before it had become permanently compressed. The operation was not repeated, neither was any more medicine given. The patient recovered the use of nearly the whole of that lung, and was in good health some years after, and then I lost sight of him. The details were given in a paper read at the College of Physicians in 1837.

"It was the result of this operation which first led me to think that an early one might be beneficially performed in laryngitis." 62.

Dr. Wilson relates three cases.

*Case* 1.—E. S. aged 46. Ill ten months, with cold, hoarseness, &c. Now complains of great difficulty of breathing, On attempting to go to sleep, a loud hiccough comes on, with great difficulty of breathing, and a sense of suffocation as if choked with wind. She points to the larynx as the seat of her sufferings. On the 10th, she had occasional paroxysms of stridulous breathing, and made such a noise that it was necessary to remove her from the ward. "Soon after, the stridulous noise returned with more intensity, and continued so till three o'clock this morning, when she became somewhat delirious, then comatose, and at seven was covered with a cold clammy perspiration; at eight her countenance was cadaverous, breathing about twice a minute, with acute stridulous sound: the larynx was immoveable during the attempts at inspiration. On applying the stethoscope to the larynx and chest no air could be heard to pass. The pupils were contracted almost to points, and insensible to a lighted candle; she looked like a person after taking a large dose of opium, but she only had had ♏xv. of the tincture in the early part of the night.

"The resident medical officers made a small opening through the integuments, just sufficient to admit a large trochar to pierce the crico-thyroid membrane, and pass into the larynx; the stilette was then withdrawn, and the canula properly fixed, when air was instantly heard to rush into the trachea and lung; though prior to this she had evidently ceased to breathe. The respiration gradually resumed its force and frequency. The pulse, which before had been intermittent, became steady, and increased in frequeny. The countenance lost its livid appearance, and the whole surface became covered with a warm perspiration."

Consciousness returned. It was necessary to substitute a curved tube for the straight one, but on the 22nd or 23rd, all tubes were dispensed with. For some time she breathed through the opening, which then closed, but would often be forced open by a paroxysm of coughing. On the 17th November, the wound closed finally. On the 21st January, she was discharged, and now, three years after the operation, she is well, with the tone and power of voice natural.

The *second case* was that of a man, aged 27, who had been ill six days with cough, hoarseness, &c. On admission, at one P.M., the face was livid and pale, with raucous breathing, and threatened suffocation, paroxysmal cough, vesicular respiration inaudible in any part of the chest, but raucous sounds distinct.

The symptoms grew worse, and in the afternoon laryngotomy was performed with immediate relief. The canula of a curved trochar, which was employed, was taken out for some hours on the next day, 16th of November, and it was finally removed on the 18th December. On the 27th January he was discharged. At the end of two years, his voice continues raucous.

Dr. Wilson remarks, in reference to these cases;—"The one may be properly classed as chronic, while the other may be as justly regarded as acute laryngitis. One of nine months' the other of six days' duration. In both the crico-thyroid membrane was pierced by a trochar. In the first, a straight or common one was used: in the other, I modified the straight into a curved trochar: for, in the first case, though the straight one answered equally well for performing the operation, yet the straight canula caused irritation at the back part of the larynx, and the open end of the tube itself was thus liable to be obstructed; from these defects, the curved one was free.

"So that, generally, in such cases, when an operation may be required, I should give a preference to this over the other ways of performing it, because the part is well marked by the thyroid and cricoid cartilages, and it is free from any large vessels. The wound thus made is the smallest possible, being only just sufficient for the tube itself; a point of some importance, as it lessens the risk of hæmorrhage from the wound escaping into the lungs, and accelerating death. Such a case I lately saw, where death took place in half an hour after tracheotomy had been performed on a child. Lastly, having made the perforation with a curved trochar, which is soon done in thin adults, when the part is not ossified, there is no further difficulty or delay in introducing a tube, for that is already done when the stilette is inserted.

"These observations are more applicable to adults than to children; for with the latter the difficulties become greater as they are younger. In these, the trachea and larynx are less prominent, and too pliant to be held firm; the parts covering them are soft, and bleed freely, while, at the same time, the struggles of the infant add to these difficulties."

Dr. Wilson relates two other cases in which the operation gave relief, but was not successful. The second case was one of croup. The child survived the operation ten hours. On examination of the body, it was found that a white false membrane lined the larynx, trachea, and bronchi to their second divisions. Beyond that the mucous membrane was red and vascular, and there was infiltrations of blood in the lungs, particularly in their lower lobes. Dr. Wilson concludes with the observation:—"Thus croup, to which children are liable, differs from laryngitis, which attacks adults: the first involving all the air-tubes, (the trachea and bronchi,)—the latter, at first at least, only the larynx, so that an opening can be made below the seat of disease in laryngitis, which cannot be done in croup.

"In concluding, it may be as well to notice, that when the canula is taken out to be cleaned, it will often be found lined with not merely an inspissated but even indurated crust, so considerable as to have diminished the calibre of the tube, and impeded respiration. The tube, when in warm water, may be freed from this by the feathery end of a quill. To clear it of mucus, when in the larynx, a more slender feather may be used. It will frequently be easier to return the curved canula, by first oiling it, and then introducing it with the end pointed upwards, and when within the opening to turn it down. A blunt probe made, like the stilette, to pass just beyond the open end of the canula, may also at times be of service in returning the tube."

### VII. Peculiar Symptoms affecting an entire Family, and terminating in Death. By *John Wilson*, M.D. Physician to the Middlesex Hospital.

The cases related in this paper are certainly most remarkable, and, in their nature most obscure.

It appears that on New Year's day, Arzoni, a Neapolitan, and manufacturer of ultramarine, was taken with griping pains and purging, which never ceased till death. He was sick at times, but never vomited. The motions were offensive and black. He had frequent cold fits through the day, followed by much fever. His joints, from the beginning, were swollen, not red, but so painful that he could not move on being put into a warm bath. His age was 47, and he died on the 20th January.

On the 2nd, an infant child of Arzoni's, and the mother, an Englishwoman, were seized with violent pains at the crown of the head, and a desire, with inability, to go to sleep. The infant died. The case of the mother, as a sort of type of the rest, may be detailed.

"During the night she lost her consciousness. Her bowels, from the first, were affected with severe pain, and she felt a frequent desire to evacuate them, when, at times, nothing but fætid gas, like that of rotten eggs, came away; at other times, the matter passed was like putrid and very offensive flesh. Three days after being first seized, she lost the use of her limbs, and had pain in all the joints. On the sixth day, œdema of the feet, legs and thighs came on. The urine was scanty, very high-coloured and offensive. The water, which from the first flowed from the mouth, had a 'cankery' taste, the glands of the neck and lower jaw were tender, and the eyes watered. The discharge of water from the mouth and eyes still continue; tongue very red, clean and transversely fissured. Gums vascular, quite clean, not swollen, but rather contracted. Abdomen large and also tympanitic. Thinks she may be four months gone in the family-way. Complains of general soreness, debility, and lowness of spirit.

"Next day after admission, the urine was brown, very alkaline, and had a white ropy sediment. She still has the peculiar taste in the mouth, but most marked under the tongue, of which taste every thing she takes seems to partake. Exquisite sensibility and soreness over all the body. Says the motions are of a better colour than they have been at any time previously. She and also both of the children now wished for some acid drink, which was given them, but her urine continued for some time afterwards very alkaline. She never rallied, but continued greatly depressed both in mind and body.

"A few days afterwards, the legs and thighs became erythematous and shining, the œdema increased and pitted. Purging frequent, and, at times, motions passed involuntarily after taking fluids. No appetite, and every thing turns sour on the stomach.

"February 8th.—For two days, itching of the whole body has taken place of the exquisite sensibility and soreness. There is a waved erythema over the back and abdomen, the skin being variegated by alternate red and white streaks. The erythema of the legs continues, though she has somewhat more use of the limbs. —Complains of thirst, and again wishes for acid drink; urine afterwards became neutral, but she became more feverish and restless. Mouth and eyes continued to water. A troublesome, dry, hacking cough came on, and continued, which prevented sleep, and caused severe pain in the head. Purging recurred, and she was obliged to keep a bed-pan under her; when the purging ceased for a time, the motions were of a dark drab colour, as they have generally been.

"The cuticle of both legs broke, and, after the discharge from them had continued for some time, the swellings had much diminished and altogether subsided afterwards; the urine increasing at the same time. Intellect has always been perfect. Often has only been able to take arrow-root and jelly.

"February 25.—Mother's pulse 72, fœtal pulse 130.

"March 15th.—Gave birth to a very small infant, which only lived twenty-four hours; its length was 14¼ inches, weight 2½ pounds. From this period Dr. Ashburner continued to see her. The pain over the abdomen remained the same as before delivery, and different from any she had felt after a confinement. Puerperal fever supervened, and on the 21st March she died.

"*Inspection, forty-eight hours after death.*—A considerable quantity of turbid fluid in both sides of the chest. Adhesions of the right lung and upper part of the left, which last had tubercular depositions at the apex. Much clear fluid in the pericardium. Abdomen contained a large quantity of turbid fluid mixed with pus and shreds of lymph. Stomach and intestines very much distended with air, but no morbid traces found in them. Liver pale; and throughout the entire body there was a great deficiency of colour. Some coagula in the large vessels. Uterus had not contracted to the size it usually does in the time elapsed since delivery. Spermatic veins of the right side of the uterus thickened, and filled with a fibrinous clot, extending up to the vena cava." 78.

*John Arzoni,* aged 11, was seized on the 14th January. He was admitted on the 26th, and died on the 11th February. The symptoms and progress of the case were very similar to those already described in the mother's. One circumstance deserves mention. For several nights, though the temperature was 10 or 15 degrees below the freezing point, he lay with only a sheet, and hardly that, over him.

*Inspection.*—"Œdema of the legs: all the muscles remarkably stiff and pale. Tissue of both lungs infiltrated with black blood, particularly the posterior parts, and so heavy as to sink in water. The margins of both lungs were emphysematous. The cavities of the heart contained very black, soft coagula, but without any fibrine. Stomach empty, much corrugated, and general surface pale, but the depressed parts of the folds were of a pink colour, and about the large arch in one of the depressions was a small excavation, narrow, red, and one third of an inch in length, filled up with a black coagulum; when washed, the excavation had an appearance somewhat like an ulcer in the process of healing. Near the same part, and within the space of four square inches, were three or four much smaller spots, similar to the above, but without coagula. Not far from the same part was a longitudinal depression, less deep than the first, and much paler, about ¾ths of an inch in length." 80.

Intestines generally pale. Brain presenting no morbid appearance.

*Mary Ann Arzoni,* aged 5, was attacked at the same time, with the same symptoms, as her brother. She was admitted on the 26th of January, and died on the 19th of June. The symptoms, though more chronic, were of the same character as in the other instances. She would ask to be turned in bed 200 or 300 times in a night. During the last few days of her life, she lay without any bedclothes over her.

The following summary of these cases will be read with interest:—

"The death of the mother was accelerated by parturition, and examination after death showed very extensive disease in both the abdomen and chest, yet probably differing considerably from what might have resulted had parturition not occurred, and had she died, like the children, of the affection common to all of them, and not been sooner carried off by a new disease supervening on the original one.

"The abrasions or ulcerations in the sulci of the stomachs of the children have been noticed; but they were so slight, that in ordinary cases they might have been overlooked, without attention being particularly directed to the inspection of that organ. Neither the mucous membrane of the intestines, nor other parts of the abdominal viscera, showed any particular change from the normal state.

"But the organ by which death finally entered, appeared in these two to have been by the lungs; for the blood at last seemed to have infiltrated into their tissue, rendering portions of them dark, and so heavy, yet not hepatized, as to sink in water, and resembling considerably the state of the lungs of those who died of the spotted fever in 1837, and which I have described in the Medical Gazette as non-circumscribed pulmonary apoplexy, caused by the blood becoming so altered as to escape from its proper vessels into the tissue of the lungs, thus rendering portions of them (as in these) black, and so heavy as to sink in water. Yet these parts were not circumscribed by healthy lung, but they gradually shaded off from the heavy dark parts to the permeable portions.

"In general the treatment of this family was merely palliative. Among the few trials had recourse to in medicine, I used the saline treatment for spotted fever, but without any apparent advantage. The same may be said of the other trials, excepting the preparation of iron, which certainly seemed to restrain the purging entirely for a length of time, and to have been otherwise beneficial in improving both the colour and strength of Mary Ann, so that she was even able to turn herself a little. To the others iron was not given.

"In all the three patients the most prominent features of their sufferings were the general soreness of the fleshy parts as well as of the joints, exquisite sensibility of the skin when touched, and the pain produced whenever they were moved, or their positions changed, according to their so frequently-expressed wishes.

"Next may be noticed, the œdema, and particularly that of the inferior extremities, which was present in all the three. The alkaline state of the urine when admitted, and their desire for acid drink. The faces of all being pale, haggard, and so care-worn, as to give to the children the aspect of aged dwarfs, from their hollow cheeks, sharp features, exsanguined and emaciated bodies. At the same time their appetites were great, and that of Mary Ann ravenous. Yet the mother's never was good, but on the contrary, at times, it was difficult to induce her to take even some trifling sustenance. The mother at different times was much exhausted by diarrhœa, and both the children suffered from it, but less severely. The father also is stated to have been affected greatly by a disordered state of the bowels.

"During their afflictions, the intellects of all preserved their integrity, and might even have been regarded as preternaturally bright, had it not been ascertained that every one of the family was reckoned quick and clever. Towards the nurses their tempers were irritable.

"Lastly, there was the 'cankery' taste of the mother. The 'metallic' taste of the boy. The watery state of the mouth and eyes in each. The teasing, hacking, dry cough, common to all, and affecting the children particularly towards the last.

"Then their lying sometimes without any covering, even during the coldest season, and when dying objecting to even a sheet; while their flesh was so greatly wasted, and the lungs becoming less and less capable of absorbing oxygen, from the gradual infiltration of blood into parts of their vesicular tissue, diminishing proportionably the permeable portions.

All attempts made by me to trace the source whence originated these affections, resulting in the loss of life, ended only in varied and conflicting accounts." 86.

A Coroner's inquest was held on the bodies of the father and infant. Nothing was elicited, and the nature of the cases remains shrouded in mystery.

### VIII. A Case of Congenital Cataract, where Sight was acquired by Operation, at the Age of Twenty-three Years.

There have been two cases on record of congenital cataract cured by operation. The patient in Mr. Cheselden's famous case was 13 years old—in Mr. Ware's, 7.

Every body knows that Cheselden's patient thought every thing touched his eye. He could in a strong light distinguish different colours, such, as black, white and scarlet, before he was couched; but, after the operation, he did not for some time perceive any difference between them. He knew the form of things by feeling, but not by sight: and it was a long time before he gained an accurate knowledge of the difference of objects when he had acquired sight. Every thing appeared to him larger than it was: of which by comparison and experience alone he was enabled at last to form a correct judgment. He was surprised that those whom he loved most, did not appear most agreeable to his eyes, expecting that they would be the most beautiful. He had the same feeling as related to taste, and was astonished that a small picture could represent a large body, saying, "It should have seemed as impossible to him as to put a bushel of anything into a pint measure." He had also but little idea of size, thinking the whole house could not look larger than his own room.

Mr. Ware's patient could, before the operation, distinguish colours, but nothing else. He acquired sight almost immediately. On the third or fourth day he knew a letter that was shewn to him, and could point out several other things both by name and color.

Mr. Stafford's patient was an interesting little girl, 23 years old. She was admitted into the Marylebone Infirmary, June 1st, 1840, with congenital cataract, apparently capsulo-lenticular, in both eyes. She had, all her life, been only able to distinguish light from darkness. She distinguished objects, and groped her way about by touch. Mr. Stafford operated by depression. The operation succeeded perfectly. The following abrégé of the results by Mr. Stafford contains what is most interesting.

"In the case here related, the acquisition of sight was very gradual. At first all was confusion. The third week she began to distinguish objects, and to be conscious of the difference of one thing from another; and was aware when a piece of rag was waved before her eye that something was moving backwards and forwards. In a month she knew that a piece of rag was white: that her prescription card was white, and that black was opposite to white, being, according to her own expression when she was shown my hat, 'quite different.' In five weeks she was acquainted with the difference of colours; for she could describe accurately the nurse's dress, which happened to be composed of three colours, white, red, and blue. Her knowledge both of form and colour rapidly improved, for in three months she could mention any article in ordinary use, and point out the difference in these respects of each, and knew the numerous colours of the different flowers of a large nosegay.

"Form and colour are simple ideas. Distance, measurement, feature, number and time, on the contrary, require two operations of the mind; first, the simple sight of them; and, secondly, a judgment to know their difference. In distance we see several objects, and judge that one is nearer to us than another. In measurement we see the object at certain distances, and judge of the different distances of one thing from another by the number of yards, feet and inches they are apart. In feature we make a comparison between two or more people. In number we first see each thing, and then count them: and in time we know there is day and night, and that the whole comprises twenty-four hours, consequently we make a measurement by the division of it into hours, minutes, and seconds. That the patient should so soon have distinguished colours and form we can understand, because it requires only one operation of the mind: but that she should (without being taught) so soon have acquired a correct knowledge of distance, measurement, the distinguishing one person from another, number and time, is very extraordinary. Distance and feature was her next acquirement. In three months from the operation, I told her to go to the different beds of the patients in the ward. The beds I wished her to go to were, the last, the last but one, the third or fourth from her, &c. She did so without hesitation. She could also tell me the name of each individual in the beds, and could distinguish the features of each person, even if twenty people were assembled together. In rather less than six months, I thought I would try whether she had any idea of measurement. As I have stated before, I took her to different parts of a large ward, and asked her the measurement from one part to the other, at several distances. To my astonishment she answered me with great accuracy. I tried the same experiment in other rooms, with the same result. I then asked her how many things she could count: she immediately went to the mantel-piece, and counted twenty, saying, 'If you wish it, sir, I can count up to 100.' I inquired if she knew what time meant. She said, 'I know the time of day when the clock strikes, but I cannot tell what time it is when I see the clock.' These questions I repeatedly asked her, and her replies were always nearly the same. I endeavoured to find out by what operation of mind she obtained this knowledge. She could not tell me. I also tried to discover what her first impressions were on seeing the human face, and other things; her respondencies were so confused and ambiguous, that I am convinced she had formed no idea. In fact, although so correct in the faculties I have mentioned, being totally uneducated, and brought up with the ignorant, she was of weak intellect, and had never reflected beyond the little occurrences by which she was immediately surrounded." 99.

The case is well detailed.

IX. Observations on Tubercle of the Brain in Children, with a Tabular View of Thirty Cases of the Affection. By *P. Hennis Green,* M.B.

Dr. Green observes, with justice, that the subject of tubercle of the brain in children has not attracted sufficient attention. He observes, too, that it would be a desirable thing if all cases of cerebral disease were carefully noted, and the appearances after death accurately ascertained. He has known many instances of "anomalous disease" of the brain, explained by lesions of the medulla oblongata which had escaped notice. He recommends that the spinal marrow should, in all cases of fatal cerebral disease, be carefully examined—a recommendation not likely, we fear, to be acted on.

He presents us with a table, which contains the name, age, sex, symptoms, and lesions, of 30 children who died from or with tubercles of the brain.

"The ages varied between nineteen months and twelve years; 13 cases occurred at the period comprised between the ages of two and four years, inclusive; a greater number than occurred during any other three consecutive years.

"With respect to sex, 14 were boys, 16 girls.

"In 4 cases no symptom whatever of cerebral disease existed during life; in 2, the chronic symptoms were confined to periodical headache; in 2, to deafness and purulent discharge from the ear; in the remaining cases the most prominent symptoms of the chronic stage were headache, vomiting, amaurosis, convulsions, paralysis, and diminution of the intellectual faculties: the duration of this chronic stage varied from one month to three years.

"Nine of the patients died with symptoms closely resembling those of acute hydrocephalus; a few with symptoms of softening of the brain; the rest of consumption, small-pox, &c.

"The volume, number and site of the tuberculous masses varied considerably in different cases: in one case twenty tubercles were found in the right hemisphere; in another, seventeen; frequently, however, they were single."

For the table itself we must refer to the Society's volume. We pass to Dr. Green's account of the history, symptoms, and diagnosis of the disease.

Tubercle of the brain is rare in adults. But, in children, it is comparatively frequent. Dr. Green has observed it in 26 instances, out of 1324 cases of acute disease, or in 1 case to every 51 of such.

*Age.*—If we may judge from 75 cases, the age at which this disease most frequently occurs appears to be from three to seven years, inclusively.

*Number.*—They are often single—often numerous. In one case there were 20 in the right hemisphere—in another 50 in the cerebrum and cerebellum.

*Volume,* varies from the size of a small nut or bean to that of the double fist.

*Seat* various, most ordinarily in the substance of the hemispheres. In the 30 cases contained in the table, the tubercular deposit existed eleven times in the hemisphere of the cerebrum; nine times in the cerebellum; seven times in the cerebrum and cerebellum together; and twice in the cerebellum and pons Varolii, together. But Dr. G. has notes of two cases in which the tubercle was confined to the pons Varolii.

*Attendant Lesions.*—"In many cases, even when the tubercle is of considerable size, we cannot discover the slightest change in the surrounding nervous substance, or in the neighbouring membranes. The gradual development of the tubercular mass seems to pass unheeded by the central nervous system. In other cases the membranes adhere to the cortical substance, over the site of the tubercle, and are more or less infiltrated and thickened. Sometimes, when the tubercle is large, the convolutions are flattened or completely effaced. The colour and consistence of the nervous substance, immediately surrounding the tubercle, present a great variety of modifications. It may be slightly injected and softened to the depth of a few lines only; or the softening of the nervous tissue, with or without injection, may extend to the central parts of the brain. In some cases nearly the whole of the cerebellum is reduced to a mere pulp. In a few rare examples, on the contrary, the surrounding nervous substance is more pale and of a denser structure than is natural; sometimes it is soft and of a straw-yellow colour. I have never seen any appearance of abscess or of true infiltration of pus in the immediate vicinity of a cerebral tubercle." 200.

There are other lesions, more complications than consequences.

*Cause.*—Dr. Green cannot satisfactorily account for the peculiar tendency of young children to this affection. The first dentition is its principal epoch—in some cases it has followed convalescence from an exanthematous disorder—in several the patients have been cut off by tubercular disease.

*Symptoms.*—Dr. G. arranges these under two stages, the chronic and the acute.

*Chronic Stage.*—Its duration varies from six weeks to two years. He has grouped these cases under three classes:—" In the first class the disease commences with headache, and then gives rise to various lesions of sensibility or of muscular power.

" In the second class, it begins with convulsions or epilepsy, which gradually terminate in paralysis.

" In the third class the first symptom observed is paralysis of one of the limbs.

1*st Class.* " Here the disease," we quote, for we cannot render more concise, Dr. Green's description, " commences with headache, which is by far the most constant and characteristic symptom of cerebral tubercle; it formed a prominent feature of the disease in seventeen of the twenty cases.

" The headache is often very severe and of an obstinate nature, preventing the patient from sleeping at night, changing the temper, and sometimes eliciting acute cries like those of hydrocephalus. The seat of the pain is generally in the forehead, but in a few cases where the tubercular mass occupied the cerebellum, the pain was seated in the occiput, and extended down towards the neck. This severe pain is, sometimes, the only symptom which exists; in other cases, after having been present for a few days or perhaps for several months it is succeeded by other symptoms, to be presently noticed.

" The attacks of headache are occasionally associated with vomiting, which recurs on each exacerbation of the pain, and cannot be traced to any disorder of the digestive organs. This chronic or sympathetic vomiting was observed in seven cases. The bowels may be constipated at these periods, but costiveness is less frequent than vomiting, and both symptoms are much more allied to acute diseases of the brain.

" The symptoms which follow in the train of headache are extremely varied: they chiefly consist, however, in lesions of the senses, the muscular power, or the intellectual faculties. The child's temper may undergo a notable change, and the intellectual faculties may become dull, but the disturbance or loss of the latter is rarely observed, except in cases of long-standing, and towards the termination of the disease.

"Convulsions sometimes occur at irregular intervals and terminate in partial or total paralysis of one or more limbs; in other cases we merely find a weakness of certain muscles, not amounting to paralysis; the child stumbles as it walks along, and progression is much impeded: particular muscles also may be affected. Thus in one case the only lesion of the motor power observed for some time was a peculiar convulsive movement of the muscles of the eye-ball, by which it was incessantly jerked inwards. In a few cases strabismus occurs.

"The symptoms connected with derangement of the sensibility are, loss of hearing, feebleness or total loss of sight, and a diminution of the cutaneous sensibility on one side of the body. The various symptoms just noticed are seldom permanent; the headache often disappears after having existed for several months, and returns again: the strabismus and amaurosis may also disappear, but the paralysis generally persists, especially when the limbs are affected by it."

*2nd Class.* In "these cases the disease commences suddenly with convulsive attacks or an access of true epilepsy: these recur at various intervals, and gradually terminate in paralysis or coma. The convulsions may be general or partial, and are often followed by contraction of one or both extremities on the same side of the body, or the head may be drawn to one side, and remain in that position for a considerable length of time. I have not noticed the deviation of the mouth or tongue, which seems to be characteristic of acute hydrocephalus. The convulsive affections often present some peculiar features. Thus, in one instance, the disease commenced with nervous tremor of the left arm, which lasted for six weeks, and then terminated in epilepsy: in another case, several attacks of convulsions were followed by a peculiar rotatory motion of the head: in a third, they were succeeded by squinting, and a lateral motion of the lower jaw. These convulsive attacks are rarely attended, as the headache is, with vomiting or constipation of the bowels.

"Instead of convulsions, the first symptom observed may be a sudden attack of epilepsy, which always terminates, after a longer or shorter interval, in general or partial paralysis. Convulsive movements existed in twelve of the twenty cases, and epilepsy in five." 205.

*3rd Class.* The disease commences with paralysis of one or more muscles, or organs of sense. Dr. Green relates a case in point. He observes that a general summary of symptoms so diversified is impossible. But the chief and most important is headache—after that come partial or general convulsions, epilepsy, paralysis or contraction of certain muscles or limbs, change of temper, and amaurosis.

*Acute Stage.* There was such in thirteen out of thirty cases. In most cases, it consists "in a succession of symptoms of an irregular character, and more or less allied to those of acute hydrocephalus, or softening of the brain. Thus the acute stage of cerebral tubercle may commence as the third stage of acute hydrocephalus, or the symptoms of the different periods of this latter disease may run rapidly into, and be mixed up with, each other. The duration of the acute stage varies from eight hours to eighteen days." Sometimes there are general convulsions which terminate in fatal coma, or they may be so violent as to cut off the patient in a few hours. Dr. Green observes:—

"The irregularity of the symptoms which occur in the acute stage of cerebral tubercle, is, I conceive, a very important point in the history of cerebral disease amongst children. Authors frequently mention the occurrence of anomalous cases of hydrocephalus, of cases in which the first stage of the disease was wanting. Is it not probable, from what has been said, that many of these hitherto unexplained anomalies depend on the complication of acute hydrocephalus, with cerebral tubercle, or, to speak more correctly, on the fact, that the acute stage of cerebral tubercle generally consists in irregular hydrocephalic symptoms?" 207.

*Diagnosis.* Dr. Green admits its difficulty. This, he observes, "depends not only on the irregularity of the symptoms, but on the length of time which often separates the appearance of one symptom from another. A lapse of several months may occur before the headache is followed by any other sign of cerebral disease, still our diagnosis must be founded on the succession of certain symptoms. The disease, it may be remarked, almost always occurs in children who manifest signs of a scrofulous diathesis. When, under these circumstances, a child has suffered for some time from severe headache, when the headache is followed by convulsive movements, some paralytic affection, amaurosis, contraction of muscles, occasional vomiting, accesses of fever, and the train of symptoms already mentioned, and when these symptoms succeed each other at various intervals of weeks or months, we have very great reason to believe that the child has tubercle of the brain."

He has never seen independent chronic softening of the brain in children—he supposes its symptoms would be similar. With chronic meningitis it may be confounded.

"The points of resemblance are the duration of the disease, the change of temper, the occasional headache, and the contraction of muscles, which occur in chronic meningitis. In this latter disease, however, the headache is not so severe or constant: we more frequently observe irregular accesses of fever, and the only permanent lesion of the motor power, which I have seen, was a peculiar flexion of the muscles of the hand and foot.

"The paralysis, amaurosis, epileptic attacks, contraction of various muscles, are peculiar to cerebral tubercle, at least so far as my experience goes." 208.

Of *Treatment* we need say nothing.

Dr. Green's paper is a valuable contribution to pathology.

### X. Case of Local Tubercular Deposit upon the Surface of the Brain. By *Robert Dunn.*

Mr. Dunn, an intelligent and experienced practitioner, has given an interesting account of this case.

The patient was a fine boy, two years of age. He had been healthy from birth, and forward, but for four months previous to his last illness had become extremely irritable.

On the 7th of October, Mr. D. was suddenly called to him. He had been attacked with convulsive jerking of the left hand. He seemed well in other respects. On the application of mustard and hot water to the hands and feet, the jerking subsided in about twenty minutes. We need not particularise the treatment that was now or subsequently pursued, as, though judicious, it did not seem to influence, materially, the case. It was ascertained that the child had had a fall about a fortnight before. At nine A.M. next day, the jerking returned and extended to the elbows, but subsided in half an hour. He suffered off and on till three P.M. of the 11th, when he was seized with a severe attack of convulsive jerking, which was not confined to the hand and arm, but involved the whole of the left side and lower extremity in convulsive agitation, with twitchings of the eye and angle of the mouth. The attack lasted two hours. On each of the two next days, he had two fits still more severe. They were followed by profound sleep, and the side was left partially paralysed. At 1 A.M. of the 14th, occurred a violent attack; the convulsive agitation of the whole of the left side, from hand to foot was excessive. In the forenoon another fit, in which the convulsive motion, contrary to its former course, had begun first in the foot, and from thence had gradually extended up the side to the arm and hand, leaving the leg paralysed and helpless. In the afternoon another fit more severe still.

From this to the 22nd, (a week) there were no fits, but there was occasional jerking of the hand and foot, with heaviness, &c. On the 22nd, he was seized with a kind of cramp or spasm—now in the hand, then in the foot,—at other times in the calf of the leg, muscles of the thigh or side, and from which, during its continuance, he seemed to suffer dreadfully. He was afflicted in this way for three or four days, when these spasms subsided, and left him with decided symptoms of effusion. He continued almost comatose for some time, when he was again seized with screaming fits, and convulsive motions in the right arm and leg. Sometimes these extended to the left side. On the subsidence of one of these attacks, he sank, on the morning of the 15th of November.

*Inspection.*—" The scalp was pale and bloodless, like the rest of the body, which was much emaciated. The dura mater healthy. The vessels on the superficies of the brain were tinged with dark blood, but there was no subarachnoid effusion. The arachnoid cavity was natural. On the surface of the right hemisphere of the brain, under both the arachnoid and pia mater, there was a deposit of tubercular matter, in patches of irregular shape and size, but the whole occupying a surface of about two inches square. The deposit was most abundant on the surface of the convolutions, it nevertheless descended into the sulci between them,—a circumstance which proved its connection with the deep surface of the pia mater. The cortical substance of the brain in contact with the tubercular matter was reddened and greatly softened, and, on microscopic examination, evinced a nearly total destruction of the tubules in it, a great enlargement of the proper globules of the grey matter and of the pigment granules which adhere to them. The softening extended a slight way into the subjacent white matter. On the edge of the left hemisphere, corresponding to the diseased patch of the right, a slight tubercular deposit had taken place in a similar manner, producing a red softening of the gray-matter in contact, but not occupying more than half an inch square in surface. The ventricles contained more water than natural—about double—and did not collapse when laid open. The cerebral substance throughout, excepting at the diseased part, was firmer than usual at the patient's age. This firmness was no doubt owing to the compression of the fluid, which probably at an earlier period of the disease was more abundant." 217.

This case forms an appropriate pendent to Dr. Green's paper.

### XI. A CASE OF STRICTURE OF THE TRACHEA. By *W. C. Worthington,* Esq. Senior Surgeon to the Lowestoft Infirmary.

C. N. aged 49, agricultural labourer, had enjoyed pretty good health. In 1833 he contracted syphilis, for which he took mercury, " not to an immoderate extent." He now experienced cough and soreness of throat, with slight difficulty of swallowing, and decline of health. In August, 1837, Mr. Worthington first saw him. He was emaciated, feeble, had uneasiness about the throat, and made in breathing a noise like that of "a roarer." Each inspiration occupied ten seconds, the chest expanding only six times in a minute. Expiration was performed in much less time than inspiration, with much less exertion and with diminished intensity of roaring. There was violent action of the hyoïd and thyroïd muscles. Vocalisation was very imperfect, and the voice raucous. There was troublesome cough, with a copious muco-purulent expectoration, the checking of which tended in some degree, to increase the difficulty of breathing. The patient complained also of an offensive discharge from the nostrils, followed by occasional exfoliation of osseous matter, which appeared to be connected with disease of the inferior turbinated bones. When the larynx was much compressed there was pain. Slight roughness of the epiglottis felt by the finger—no stethoscopic indication of disease of the lungs.

Mr. Worthington thought that little could be done. The patient went on for nearly four years. The peculiar roaring sound and raucous voice never left him. He was generally worse when the atmosphere was damp and cold, and when exposed to the night air. In the winter months he was mostly confined to the house, but as the weather became warmer, he could, if allowed to take his own time, walk about three or four miles in the day. Whatever promoted expectoration, usually produced a temporary relief of the dyspnœa. He described the expectorated matter as having sometimes assumed an arborescent appearance. His death took place the 15th March, 1841. On the morning of the day of his death, whilst taking some bread and milk for breakfast, some particles of this food fell into the larynx, and he was suffocated in less than five minutes.

*Inspection.*—Muscles in front of neck unusually developed. Lungs moderately distended, otherwise sound. Bronchial tubes filled with viscid mucus. Bronchial glands enlarged, one calcareous. Two ounces of fluid in the pericardium.

*Trachea.*—"A singularly well-defined constriction, constituting complete stricture, was discovered just below the cricoid cartilage, the calibre of the strictured portion not exceeding that of a crow-quill, and at once disclosing the principal cause of the distressing symptoms during life. This partial obliteration of the canal was independent of any adventitious membrane, the product of either acute or chronic inflammatory action, as in croupy affections, and of the existence of any of the usual marks of inflammation. The tracheal rings, at the point of stricture, had entirely disappeared, and had been converted into a fibro-cellular tissue, whilst those below the constriction were much dilated beyond their natural circumference, and had also to a certain extent lost their elastic and cartilaginous character. The larynx, when held perpendicularly, presented a more flattened appearance than natural, owing to the approximation of the alæ of the thyroid cartilage. This altered shape may probably be regarded as a consequence of the stricture in the trachea, and it no doubt in some degree added to the difficulty of breathing. The epiglottis showed marks of having been attacked with ulceration at some former period; the only vestiges of it remaining were two or three small irregular vegetations. The lining membrane within the larynx was slightly thickened, pale, and rather thickly smeared with a viscid muco-puriform fluid, but it presented no appearance of ever having been the seat of ulceration." 225.

Dr. J. Copland adds in a note:—

"By the permission of Mr. Worthington, the trachea, sent by him with his paper to the Society, was slit open in the middle of the membranous portion by Mr. Shaw. Its inner surface presented superficial cicatrices extending both below and above the strictured portion. The cicatrized surface was smooth, although somewhat irregular, and of a serous or polished appearance; showing that the ulceration had healed long previously to death. The cartilaginous rings of the trachea were entirely absorbed from about half an inch below the thyroid cartilage downwards to the extent of about three inches. The upper part of the trachea that had thus lost the antagonising power to the transverse fibrous structure, was constricted so as to admit only a crow-quill. The inner surface of the constricted part was quite smooth. The larynx was sound; but the inner surface of its base and the commencement of the trachea presented superficial, slight, and old cicatrices. The trachea was much dilated from a little below the strictured part, to the bifurcation, and its internal surface presented the superficial cicatrices already mentioned." 226.

Mr. Worthington suggests that the tracheal disease was probably of syphilitic origin. On this point we do not agree with him. The disease of the nasal bones, as well as tracheal ulceration, were no doubt mercurial, for we disbelieve the tendency of syphilis alone to give rise to such lesions. As it happened, tracheotomy might have saved the patient, but, of course, it was impossible to say what was the exact site of the contraction. The probabilities, however, in such a case, would point to the larynx or its immediate vicinity.

XII. HISTORY OF A REMARKABLE CASE OF TUMOURS, DEVELOPED ON THE HEAD AND FACE, ACCOMPANIED WITH A SIMILAR DISEASE IN THE ABDOMEN. By *Henry Ancell*, Surgeon to the Western General Dispensary.

This is a very curious case, related with great precision and clearness, by Mr. Ancell.

Frances Massenger, unmarried, aged 52, applied at the Dispensary, May 1840. We know not how we can satisfactorily condense the following description:—

"The greater part of the scalp and face was loaded with solid tumors. Those on the scalp were externally of a very florid colour, smooth, glossy, and denuded of hair. They varied from a pin's head to a horse-chesnut in size, and from a nearly globular to an irregular flattened spheroidal form, with a tendency to assume a mammillated outline. A few tumors, perfectly round in shape, and of a violet hue, were interspersed; forming a remarkable contrast to the former, and never attaining so large a size. Their colour evidently depended upon their vascularity, vessels containing red blood being observed ramifying upon the parietes of those which were red, and larger vessels containing dark blood upon the violet ones; but their texture possessed a considerable degree of transparency, and there was, accordingly, an appearance of greater general vascularity than really existed. They were deprived of much of their colour on slight compression, but on suspending this the blood returned rapidly, so as to restore them to their natural hue. Some were sessile on broad bases. Others, including many of the largest, were appended to the scalp by short thick peduncles. One of the latter having been removed by incision, and divided diagonally, was nearly of a cartilaginous consistence. It exhibited a smooth, shining, semi-transparent texture, of a very pale pinkish hue, and was apparently homogeneous, except that a few distinct vessels, from which blood could be easily pressed, ramified through it. There was much greater vascularity in the investing skin than in the tumor itself. The scalpel employed was not rendered in the slightest degree greasy, and scarcely even soiled. The portions of the scalp from which the tumor was removed bled rather freely. One of the blue variety had been in the right ear for years, completely filling the meatus, and occasioning deafness. These tumors sometimes itched; considerable pain was excited by pinching them; and the patient's statement was, that 'just before rain they shoot and leap a good deal,' but otherwise they were free from uneasiness. Tumors of this nature covered a great portion of the hairy scalp and forehead, and numerous small ones were scattered over the face, but here they were mixed with tubercles, which differed from them in their general characteristics, as will presently be described.

"One of these tumors was re-examined after being kept about a fortnight in Goaltby's saline solution. The texture now presented more of a granular appearance; and although the integuments were very thin and semi-transparent, they formed an indistinct capsule, which could be torn from the subjacent parenchyma, leaving a very rough surface. A small portion of the substance from the interior having been opened out with a needle, placed between two plates of glass, compressed into a very thin stratum, and examined under the microscope with a glass an eighth of an inch focus, had, in the mass, an obscure cellular structure, and surrounding and attached to it were several distinct, nearly circular, nucleated globules, resembling those figured by Müller as characteristic of one variety of encephaloid disease.

"The skin of the face, neck, and shoulders had a remarkable tawny aspect, and was very coarse and rough, the roughness depending almost entirely upon numerous tubercles before alluded to, many of them extremely minute, others as large as a split pea, and of all intermediate dimensions. They were most thickly set about the nose, eyebrows, and ears. The larger had all the characters of lenticular tubercles, depending upon hypertrophy of the dermis, since they were smooth and very hard, of the same colour as the surrounding skin, and no sebaceous matter could be pressed out of them. Most of the smaller ones were manifestly follicular elevations, such as accompany other cutaneous diseases; they were a few shades whiter than the surrounding skin, resembling acne punctata without the black point, and exuding on pressure a white substance, similar to curdled milk." 230.

She was a native of Leicestershire, worked in the fields, had first seen the disease at 14 or 15 years of age, but had found a great many small tumors grow during the last year or two. The late Mr. Rose, of St. George's Hospital, extirpated some of the tumors. In July, 1826, she applied to Mr. Bryant, who, at one sitting, removed sixty. They were then less firm, and, on making a longitudinal incision their contents were easily turned out. Within twelve months, the tumors were all reproduced. About five months previously to her application to Mr. Ancell, she discovered a hardness in the abdomen. On examination, an uneven tumor was detected in the right hypochondrium, where, at times, she experienced pain.

After a time ascites occurred, followed by anasarca of the lower extremities. The anasarcous limbs inflamed and sphacelated, and she sank exhausted in February, 1842.

Her grandmother was affected with similar growths on the head. Her mother had a large one in the same place, and died dropsical at the age of

79, leaving a large family. A younger sister has had a mammary tumor extirpated. Her eldest sister, aged sixty-four, is free from the disease. She has had fifteen children, most of whom are married; two of her daughters have each twelve children ; and she has more than forty grandchildren and four great-grandchildren living; the whole of this branch of the family being exempt. Another sister, aged sixty-two, is affected with a large crop of tumors on the head, forehead, temples, and about the ears. They resemble the larger vascular tumors in the present case. She is the mother of a large family, several of whom, including two sons, are similarly affected. In no instance has the disease been transmitted by the males of the family.

*Inspection.—The peritoneum* "was generally opaque, but with a shining surface. The portion lining the abdominal parietes was very considerably thickened and indurated: it was also studded with myriads of tumors, projecting into its cavity, many of them not greatly varying from the size of peas, and the whole producing a yellowish granulated appearance. The peritoneal surface of the diaphragm was thickened and studded with similar tumors, either in patches from the size of pins' heads to that of small peas, closely huddled together and compressing each other; or more thinly set, very minute, white and semi-transparent."

"The minute specks were sessile, and in many instances scarcely, if at all, raised above the surface; but all the larger ones tended to become pendulous, and some were completely so, hanging by short necks." 234.

Mr. Ancell thinks there can be little doubt that the affection was seated in the cellular aspect of the peritoneum.

In the *great omentum*, from which the fat had been greatly absorbed, there were numberless granules about the size of pin's heads, with larger masses generally of a globular form, nearly white, or looking like schoolboys' veined marbles.

*In the mesentery* were much larger, and more irregular masses. A few mesenteric glands were slightly hypertrophied, and the surface of the intestines was speckled with the minute granules.

"The peritoneal coating of the superior surface of the liver was thickened, opaque, and free from the deposit: but attached to the anterior edge of this viscus, in a manner suspended from it, and extending beneath the right lobe, displacing and pressing the gall-bladder downwards into Glisson's capsule, a very large mass was found, weighing perhaps two pounds. It had evidently been deposited between the layers of peritoneum at the anterior edge of the liver, since the membrane was continuous from the surface of the organ over the tumor, the whole of which it enclosed as a capsule. The thin edge of the liver was however spread to a considerable extent over the upper and anterior parts of the surface of the tumor. The gall-bladder was stretched along its under surface. Two or three small deposits were also observed near the larger mass, but isolated in the substance of the organ, and a great number of the pendulous tumors were attached to the loose cellular membrane which surrounded these parts and to that which constitutes Glisson's capsule. The divided surfaces of these smaller tumors presented an appearance similar to those of the vascular tumors on the head and face." 237.

"The large tumor was of an irregular ovoid form, with a nodulated surface. It possessed a very firm texture. The scalpel with which it was divided diagonally was not soiled in the slightest degree. The tints presented by the cut surfaces were extremely varied, green and greenish-yellow predominating. It was nearly white, and almost cartilaginous at its centre, and there were distinct fibrous radii, of irregular dimensions, proceeding from the centre towards the circumference. The remainder of its substance was made up of large lobules, varying in size, and these again presented an indistinctly cystiform aspect in their interior and outline." *Ib.*

The tumor could not be called highly vascular.

Much limpid fluid in the ventricles and between the membranes of the brain. A tumor of the size of a pea, and another much larger, in the substance of the uterus.

"Some of the profession who saw the external tumors designated them molluscum, others vascular sarcoma, and the terms scirrhus, fungoid growth, encephaloid in a crude state, albuminous sarcoma, and colloid cancer, have been applied to the internal disease. Mr. Kiernan made the section of the large mass, but declined giving it any name." 238.

Mr. Kiernan was wise in his generation. For certainly there is no one class of cutaneous complaints to which this can be fairly said to belong. Mr. Ancell makes many just observations on the nosological characters and position of the case, for which we must refer to the original. He concludes thus:—

"Upon the whole, then, it would appear, that there exists a diathesis or state of constitution subject to an aberration of the nutrition of various parts, or a particular tissue, and that the local aberration as well as the diathesis are deficient in some of the characteristics of cancer, although, from the similitude in anatomical structure of the diseased tissue to true scirrhus, attended with symptoms of cancerous cachexia, we can but suspect that, owing to causes superadded, these growths are liable to become carcinomatous and destructive." 246.

### XIII. Account of a Case of Irregular Formation of the Heart, accompanied with a Supernumerary Valve in the Pulmonary Artery. By *Theophilus Thompson*, M.D. Physician to the Northern Dispensary.

In December, 1841, Dr. Thompson visited, as a dispensary patient, A. H., unmarried, aged 38. She was sitting up, drowsy, livid, with the external jugular veins much distended, pulse rapid and feeble, impulse of heart rather weak; the first sound shorter and more flapping, the second less distinct than natural, and both sounds unattended with roughness. There was anasarca. This increased, the legs became gangrenous, and she died in the second week in January.

She had been pretty well till attacked with the Asiatic cholera, during its prevalence, after which she complained occasionally of palpitation. Two years before death she had "black fever," from which time she exhibited the lividity and drowsiness, &c.

*Inspection.* Some effusion into the peritoneum, pleura, and pericardium, and serous infiltration, with redness, of the bronchial tubes.

"The heart was larger than natural, and exhibited a circumscribed dilatation at the part of the right ventricle more immediately connected with the pulmonary artery. On making an incision from that artery along the anterior part of the ventricle, four pulmonary valves were observed, but the tricuspid valve was not visible. A second incision parallel to the first was then made at the back part of the ventricle, by which means the tricuspid valve was discovered, separating the right auricle from a cavity corresponding in size and appearance to the right ventricle in its natural condition, excepting that the valves of the pulmonary artery were not seen. It was now obvious that the two cavities just described constituted the right ventricle, which was divided into two portions by an imperfect septum. This septum was composed, not of a uniform fleshy wall, but of decussating and hypertrophied columnæ carneæ; some of which separating from each other near the base of the ventricle, left an aperture of communication about an inch long, and half an inch broad. Nearer the apex there were other small interstices amongst the columns, through which, although by a tortuous and difficult course, a small quantity of blood might probably have passed from one cavity to the other. The arterial chamber of the right ventricle was rather less spacious than that adjoining the auricle; the connecting orifice was partially covered by one of the divisions of the tricuspid valve.

"The walls of the left auricle, and of both the ventricles, were of natural thickness; but the right auricle was twice as thick as the left, and with very large and prominent musculi pectinati. The columnæ carneæ of the left ventricle appeared singularly small when compared with those of the right. The four valves of the pulmonary artery were found on admeasurement equal in size. Each of the valves was well developed, furnished with a corpus sesamoideum, and about nine-tenths of an inch in diameter. As each of the valves was of natural size, an additional valve was rendered necessary by the preternatural magnitude of the pulmonary artery, the circumference of which exceeded that of the aorta by nearly an inch.

"The divided valve, and that adjoining, rest on a fleshy column, nearly an inch in thickness.

"It may be well to add, in reference to the chamber of the right ventricle contiguous to the auricle, that the portion of the tricuspid valve, near the coronary vein, is attached by tendinous cords, an inch long, to hypertrophied columns, forming the inner side of the aperture connecting the ventricular chambers. The other portion of this segment, and the adjoining segment through which the incision was made, possess tendinous cords only half as long, and attached to columns of average size. The intermediate portion of the tricuspid valve has cords of intermediate length, all of which are attached to one projecting thickened column, excepting that from the edge of the valve, covering the connecting isthmus, a few cords pass to the inner side of the ring, within half an inch of one of the semilunar valves." 250.

Dr. Thompson observes that irregularities in the number of the semilunar valves are rare. He refers to several, and to the sort of law, laid down by Meckel—that if augmented in number they are diminished in size, and vice versâ. In the present case this was not so, a perfect valve being added, while the supplementary ventricular cavity is interesting. This probably congenital malformation was of little moment till the muscular energy was impaired, when the heart suffered embarrassment, in consequence of the indirect course of the blood through its right cavities; and when the supervention of bronchial affection interrupted the circulation of blood through the lungs, a livid complexion, œdema, and gradual exhaustion of strength, were the natural results.

### XIV. Notes of a Case of Petechial Cow-pox, with Observations on the Development of the Hæmorrhagic Diathesis. By *George Gregory*, M.D. Physician of the Small-pox and Vaccination Hospital.

May 19, 1842, Mary Ann Webb, aged 4, a fine child, "was vaccinated at the Small-Pox Hospital, by Mr. Marson, in five places on the left arm. The child was at the time apparently in perfect health. Her brother James, aged six, and her sister Jane, aged one year and a half, were vaccinated at the same time. The lymph for all the three children was supplied from the same source, an unexceptionable eight-day vesicle.

"On Sunday, May 23rd, the mother first perceived that the arm of the child Mary Ann presented some unusual characters. It appeared to her more inflamed than the arms of the other children, and she noticed at the same time some spots on the child's face. The child, however, made no complaints, and in all other respects enjoyed its usual state of health. On Thursday, May 6th, being the eighth day, the child walked to the hospital and back without feeling fatigued. The vesicles on that day appeared dark, as if filled with blood; the areola was of a mahogany colour, obviously from ecchymosis; and numerous petechiæ were dispersed over the whole body, more especially the face, neck, and arms. There were several patches of ecchymosis on the tibia. The child's appetite and sleep were unimpaired.

"On the following day, Friday, the 27th May, I first saw the child. The outer portions of a large areolous circle had assumed a yellowish tint, while the inner portions were still of a dark mahogany colour. The vesicles themselves were jet black. It was obvious that there had been extensive ecchymosis around the incisions, which was in process of absorption. The petechiæ over the body were numerous. On the left temple there was a very large extravasation of blood, owing to a slight bruise which the child had received. There had been some bleeding from the left ear, and a few drops of blood had escaped from the nostril. The child's general health was good. The bowels had acted freely from medicine taken the preceding day. No blood was perceptible in the motions.

"The brother and sister of the child were passing through the cow-pox in a perfectly normal manner.

"Dr. A. Todd Thomson, Dr. Quain, Mr. Davis of Hampstead, Mr. Porter, and other gentlemen, visited the child on this and the three succeeding days. The ecchymosed state of the arm, and the petechiæ, declined with the decline of the cow-pock. On Friday, June 3rd, (the sixteenth day of vaccination,) all hæmorrhagic appearances had ceased. Two scabs had fallen off, leaving good cicatrices. Three others, hard, and of a jet black colour, still adhered. The child was in perfect health."

The child had cut its teeth with fits, had not passed through measles or scarlet fever, and had never been seriously ill.

Dr. Gregory makes some interesting observations, tending to the point that the hæmorrhagic diathesis in this case was the result of the introduction of the vaccine as of any other morbid poison. Dr. Gregory had never previously seen such a case. But Mr. Gardener, of Great Portland street, a very intelligent practitioner, saw a case of this kind some years ago in Westminster. In the Museum of Guy's Hospital there is a wax model, (No. 2,705) described as "The arm of a young man affected with purpura, consequent to vaccination." In this, however, the eruption is of an anomalous character, in large circular patches, and the vaccine vesicles are not filled with blood. Dr. G. would rather characterize this as "a case of adult vaccination performed in a very unhealthy state of the system."

Of petechial small-pox Dr. Gregory has seen many instances. It is generally fatal. He notices the important distinction that subsists between cases of petechial cow-pox and those wherein the vesicles generally or partially fill with blood from the violence of the local inflammatory action. The latter he has frequently witnessed.

### XV. ON ACUTE ULCERATION OF THE DUODENUM, IN CASES OF BURN. By *T. B. Curling*. Lecturer on Surgery and Assistant Surgeon, London Hospital.

Mr. Curling observes that, so obscure are diseases in the duodenum, that authentic instances may throw light upon them. He relates twelve cases. We will not notice all, but only the more leading ones.

CASE 1.—*Extensive Burn—Ulceration of the Duodenum—Fatal Hæmatemesis.*

M. A. Fox, a girl aged 11, was brought to the London Hospital, May 9th, 1841, on account of a severe burn on the chest and both arms, the skin of which was extensively destroyed. She had apparently been going on tolerably well until the 27th, when there occurred profuse hæmatemesis. She afterwards repeatedly ejected blood from the mouth, and also passed some by stool, and notwithstanding the remedies employed, expired in fifteen hours after first vomiting blood.

*Inspection.* "In the duodenum, at the distance of an inch from the pylorus, there was a circular ulcer about half an inch in diameter, and its edges slightly elevated, which had extended through all the coats of the intestine, the bottom of the ulcer being formed by the glandular substance of the pancreas, which was closely united to the duodenum at that part. The open mouth of a considerable-sized vessel could be distinctly seen at the base of the ulcer, apparently on the surface of the pancreas. There was no further disease of the intestinal canal, but it contained a good deal of dark-coloured blood mixed with the fæces." 261.

CASE 2.—*Extensive Burn—Perforating Ulcer in the Duodenum—Death from Hæmorrhage.*

"A fine male child, aged 4 years, was admitted into the London Hospital, Sept. 11th, 1840, under the care of Mr. Luke, having sustained an extensive burn on the neck, chest, and both arms. The case was treated in the usual way, but on the 24th, about 11 A.M., after complaining of heat and pain in the abdomen, he vomited about half a pint of blood, and afterwards continued to pass blood by stool at different periods till his death, which occurred on the following day, in the evening, after a convulsive fit. The bowels were not relaxed previously to the hæmorrhage." 262.

*Inspection.* "A large solitary ulcer was found at the posterior part of the duodenum where it passes in front of the head of the pancreas. This ulcer was of an irregular form, and three quarters of an inch in diameter at its broadest part. It had destroyed the whole of the coats of the gut, so that its base was formed by the pancreas. So slight was the connection of the margin of the ulcer to this gland, that in disturbing the parts in their removal, the border of the ulcer gave way, and allowed the escape of a portion of the contents of the duodenum into the cavity of the abdomen. The edges of the ulcer were smooth and elevated. A large blood-vessel was distinctly seen running across the base of the ulcer in a transverse direction. The anterior part of the parietes of this vessel was destroyed, so that the remains presented merely a grove or channel, which terminated near the edges of the ulcer, at the opposite sides, in open mouths." 263.

The follicles throughout the intestines were well developed.

The *third* case was one of fatal burn, with perforating ulcer of the duodenum. The patient died on the tenth day, having passed blood by stool shortly before death.

Mr. Curling relates several other cases, but as they all point to the same fact, the connexion between inflammation of the duodenum, and its consequences, and burn, it is scarcely necessary to detail them.

Mr. Curling seems to think that liability of the duodenum to suffer in cases of burn depends on some peculiar sympathy between the glands of Brunner and the skin. He argues :—

"The duodenum is furnished with peculiar glands, the true glands of Brunner, which abound in that particular part of the intestine, the seat of disease, and though their office and the nature and uses of their secretion have not been well ascertained, their size and number indicate that they must be capable of pouring out a large quantity of fluid, and that their functions in the economy are by no means unimportant. Now it is seldom that the secretions of any organ can be suddenly stopped without injurious consequences resulting, and considering the importance of those of the skin, and the continuity of this structure with the mucous surface of the alimentary canal, we can scarcely be surprised that the duodenal glands should sympathise and endeavour, by an increased action, to compensate for the suppression of the exhalation from the skin, and that the irritation consequent thereon should often lead to inflammation and ulceration." 277.

If we may trust to M. Cruveilhier, the glands of Brunner, in the duodenum are like the labial and buccal glands, salivary. Certainly glands of that description are not prone to disease. Nor does Mr. Curling offer any satisfactory proofs, drawn from dissection, of this theory. Now, however, that attention is drawn to the subject, it may be narrowly looked into. We confess that we are inclined to regard congestion of the mucous membrane as the real origo mali. Be this as it may, it is well that the profession should be alive to the occurrence of duodenal mischief in these cases. As Mr. Curling very justly observes :—

"It has been noticed by authors, that in cases of extensive burn, patients often appear to be going on well, the constitution seeming to bear up against its destructive effects, when the powers suddenly give way, and the patient rapidly sinks. In many of these cases, if inquiry had been made, it would very probably have been found that the unfavourable change had resulted from the occurrence of hæmorrhage or perforation from an ulcer in the duodenum. Indeed, in two cases which have come under my notice, the surgeon in attendance was quite unaware of there being any bleeding from the bowels, the nurse having neglected to inform him of the alteration in the appearance of the stools." 276.

XVI. OBSERVATIONS ON A PARTICULAR FORM OF ENCYSTED TUMOR, WHICH OCCURS IN THE NECK, BUT IS NOT NECESSARILY CONNECTED WITH THE THYROID BODY. By *Benjamin Phillips*, F.R.S. Surgeon to the St. Marylebone Infirmary, &c.

Mr. Phillips describes what Maunoir of Geneva had described before as *Hydrocele of the Neck*, and has been described also by Mr. Hill, of Dumfries, Dr. O'Beirne, and others. The following is his summary drawn from the cases of all.

"The cases which I have detailed, as well as those described by the authors I have named, justify me in stating, that there is a class of unilocular or multilocular encysted tumors developed in the neck, which contain a serous fluid, varying from a light yellow to a deep coffee-colour; that in its nature this fluid is albuminous, coagulable by heat; that those tumors are generally developed, quite independently of the thyroid body, though in their progress they may become intimately connected with it, but that the connection does not usually involve any change of structure in that organ; that they are most frequently developed at, or after, the middle period of life; that they occur almost indiscriminately in both sexes; that they are almost always of slow growth, and that they often attain a large size; that they do not usually prove troublesome, until they are of sufficient size to interfere with respiration or deglutition. That the treatment by simple puncture, so as to evacuate their contents, is usually insufficient to cure the disease, because the opening is apt to close up and the cavity to refill; that puncture, with injection, has not succeeded better, and for this reason, either the fluid is too stimulating, and produces violent inflammation, with spasmodic action of the organs of respiration and deglutition, or it does not exercise sufficient action to modify the surface of the sac; and it is not easy to ascertain the just medium; that the treatment by making an incision of sufficient extent to expose the greater part of the interior of the sac, and stuffing the cavity, has been followed by too much local and constitutional irritation to render it prudent to adopt it as an ordinary method of cure; that the plan which has succeeded best is that to which Mr. Hill, of Dumfries, resorted, and which has been employed by M. Maunoir, Dr. O'Beirne, and Petrale, namely, puncture and seton. Whether an ordinary tent passed through the long diameter of the sac, after puncture, might not answer the purpose equally well, we have not yet the necessary experience to determine, but I see no reason why it should not succeed." 303.

He relates, himself, two cases. In the first, the cyst discharged itself through a puncture, made with an exploring needle. The fluid, in the first instance, was thin and bloody, then became bright red, and reduced the strength of the patient as well as the size of the tumor. Next the discharge became sero-purulent, and lastly purulent. It stopped under the influence of time, *or* quiet and swallowing ice. The patient recovered, the tumor having lessened very much, but the thyroid body remaining enlarged.

In the second case, the tumor cracked spontaneously, and about three pints of reddish serous fluid escaped. On the following night, three pints more escaped. A sero-purulent discharge flowed for many weeks, when she recovered, an inconsiderable enlargement of the thyroid body remaining. Twelve months afterwards, there had been no re-accumulation of fluid in the sac, but the enlargement of the thyroid body still continued, and a small fistulous communication with the sac remained. We fancy that this affection is not very uncommon.

